# LPTA: Location Predictive and Time Adaptive Data Gathering Scheme with Mobile Sink for Wireless Sensor Networks

**DOI:** 10.1155/2014/476253

**Published:** 2014-09-03

**Authors:** Chuan Zhu, Yao Wang, Guangjie Han, Joel J. P. C. Rodrigues, Jaime Lloret

**Affiliations:** ^1^College of Internet of Things Engineering, Hohai University, Changzhou 213022, China; ^2^Instituto de Telecomunicações, University of Beira Interior, 6201-001 Covilhã, Portugal; ^3^University of ITMO, Saint Petersburg 197101, Russia; ^4^Integrated Management Coastal Research Institute, Universidad Politecnica de Valencia, 46022 Valencia, Spain

## Abstract

This paper exploits sink mobility to prolong the lifetime of sensor networks while maintaining the data transmission delay relatively low. A location predictive and time adaptive data gathering scheme is proposed. In this paper, we introduce a sink location prediction principle based on loose time synchronization and deduce the time-location formulas of the mobile sink. According to local clocks and the time-location formulas of the mobile sink, nodes in the network are able to calculate the current location of the mobile sink accurately and route data packets timely toward the mobile sink by multihop relay. Considering that data packets generating from different areas may be different greatly, an adaptive dwelling time adjustment method is also proposed to balance energy consumption among nodes in the network. Simulation results show that our data gathering scheme enables data routing with less data transmission time delay and balance energy consumption among nodes.

## 1. Introduction

With the enormous development in the field of embedded computing and wireless communication technology, wireless sensor networks (WSNs) nowadays are more applicable and practicable than the WSNs in the past were. A wireless sensor network is composed of hundreds or thousands of distributed sensors that monitor their interesting surroundings and report sensed data to a static base station or sink through multihop relay. Typical applications of WSN include target tracking, environment monitoring, military surveillance, health monitoring, natural disasters forecasting, and so forth [1–3]. Sensors with limited energy are usually left unattended after the initial deployment. And the environments of the deployment area are often harsh and involve obstacles, which makes sensors battery replacement infeasible. Hence, it is expected to minimize and balance energy consumption of sensors to prolong the network lifetime. It has been proved that in a static network, the sensors deployed near the sink exhaust their energy faster than those far apart due to their heavy overhead of relaying messages, and this is the so called “hot-spot” problem [4]. In addition, node failure or malfunction may cause energy holes in the deployed area, and the network connectivity and coverage around the sink may not be guaranteed [5]. Therefore, unbalanced energy consumption causes network performance to be degraded and network lifetime shortened.

Recently, various new strategies that use mobility attributes of elements in WSNs have been investigated to reduce and balance energy consumption of sensors [6, 7]. In this paper, we consider utilizing a mobile sink to proactively collect data generating from sensor network, and this strategy is also favored by many researchers [8–10]. When the sink moves in the network, the role of the “hot-spot” rotates among sensors [11], resulting in balanced energy consumption among nodes. The effectiveness of using mobile sinks to gather data has been demonstrated both by theoretical analysis and experimental study [12–15].

Generally, when a mobile sink is adopted to collect data all over the network, it should consider the following two requirements.

- *Low Data Delivery Latency*. Cheap sensors deployed in the monitored environment are often equipped with limited resources, that is, energy, memory space, and so forth. And the speed of a mobile sink is relatively slow compared with the speed of wireless wave. These features may cause a long data transmission delay, which will lead to sensor nodes out of memory and packets lost. Therefore, reducing data delivery latency is necessary, especially in time-sensitive applications.
- *Less Control Message Overhead*. When a sink moves, it must broadcast its current location to the sensor nodes in the network repeatedly. The broadcasting among sensors consumes a large amount of energy, resulting in rapid energy depletion. Due to the limitation and the preciousness of the energy resource, it is particularly important to reduce the energy consumption of the control overhead among sensor nodes.

In this paper, we propose location predictive and time adaptive (LPTA) data gathering scheme with mobile sink for wireless sensor networks, which extends the lifetime of the network with less sink's location updating overhead and reduces the data transmission latency. The data gathering process is composed of multiple data gathering periods. Each data gathering period consists of three phases: loose time synchronization phase, date collection phase, and data gathering period ending declaration phase. The following primary features make our LPTA scheme differentiated from other existing data gathering algorithms [8, 9, 16].

*(i) LPTA Needs No Location Updating Information for the Mobile Sink*. Many existing data gathering algorithms, for example, ALURP [8, 9], need to broadcast the mobile sink location information during data gathering process. Our proposed scheme is based on a loose time synchronization mechanism among sensor nodes and the mobile sink. Therefore, each node in the network is able to calculate the position of the mobile sink based on their local clock. It significantly reduces the control messages overhead and makes the data reporting at any time possible for every sensor node.

*(ii) LPTA Is an Adaptive Algorithm in Terms of Sink's Dwelling Time*. In many scenarios, the deployed environments are not ideal, and often involve obstacles. The deployment of sensor nodes in such environments is nonuniform. Additionally, the frequentness and number of the events of interest may be different for different part of the deployment area during different periods. In LPTA, according to the quantity of the data generated from different area of the network, the mobile sink's dwelling time at different sojourn points can be adjusted adaptively, resulting in more efficient energy consumption of nodes.

The contributions of this paper can be summarized as follows.

(*1)  LPTA Utilizes Location Prediction to Reduce Data Transmission Delay and Location Broadcasting Overhead*. The loose time synchronization at the beginning of each data gathering period and the mobile sink's fixed moving track with constant speed make the calculation of mobile sink location possible.

(*2)  LPTA Avoids Getting Sensor Nodes out of Memory*. When there is data to report or relay to the mobile sink, sensor nodes can calculate the location of mobile sink and send out the data immediately. Therefore, sensor nodes need not cache the data for a long time and wait for the sink moving to neighboring scope.

(*3)  LPTA Takes Obstacles into Accoun, and Can Adjust the Sink Dwelling Time at Sojourn Points during Data Gathering*. The criteria for the time adaptation is based on the quantity of history data generated in each area. When gathering data, the mobile sink will dwell longer time in the area with more data to send than those with less, resulting in the average relay hops decreased and the energy efficient for data transmission.

As the implication of of LPTA abbreviation, our LPTA mainly focuses on two strategies:

1. mobile sink location prediction,
2. dwelling time adjustment of mobile sink.

During the process of data reporting from nodes to the mobile sink, shortest data routing protocol or other existing routing protocols can be adopted, as long as data can be relayed to the mobile sink based on location information. To simplify our scheme and focus on the two strategies of LTPA, we use shortest path routing protocol as the data routing protocol.

The rest of this paper is organized as follows. We present related work in Section 2. The network model and problem statement of our scheme are given in Section 3. Then, the moving strategy of the mobile sink is presented in Section 4, and the data reporting process of nodes towards the mobile sink is described in Section 5. And in Section 6, the performance of our protocol is analyzed and compared with that of the adaptive sink mobility scheme (*Adaptive*) [16] in terms of data transmission latency and energy consumption through simulations. Finally, the conclusion is given in Section 7.

## 2. Related Work

Here, we briefly summarize some of the related works on data gathering mechanisms in WSNs. According to the main purpose of these works, the existing protocols can be classified into three categories:* extension of network lifetime*,* reduction of sink's location updating overhead,* and* reduction of time delay of data reporting*.

### 2.1. Extension of Network Lifetime

To alleviate the influence of “hot-spot,” a number of research works on prolonging the lifetime of WSNs by using mobile sinks have been proposed. Wang et al. [17] explored sink mobility to prolong the lifetime of sensor networks. They gave a linear programming formulation for the joint problems of determining the movement schedule of the sink and the sojourn time at different points in the network. Their proposed routing scheme can work only in a grid network topology. Luo and Hubaux [15] proved that in a circle topology, to achieve the maximum network lifetime, a mobile sink should rotate on the periphery of the network. A joint mobility and routing strategy with a combination of periphery moving and round routing was proposed. Then, by assuming Manhattan routing, Lee et al. [10] obtained the similar conclusion. They also proposed a heuristic algorithm for sink mobility to achieve near-optimal network lifetime. Liu et al. [18] proposed a density adjustment algorithm in order to increase the network lifetime and coverage by appropriately adjusting node density. A biased adaptive sink mobility scheme (*Adaptive*) was proposed in [16]. In order to achieve accelerated coverage of the network and fairness of service time of each region, the sink moves probabilistically, favoring less visited areas and adaptively staying longer in network regions that tend to produce more data.* Adaptive* balances the energy consumption among nodes and prolongs the network lifetime. However, because the mobile sink has to traverse all vertexes in the graph, it may cause a rigorous time delay problem in large scale networks.

### 2.2. Reduction of Sink's Location Updating Overhead

Sink's location updating may cause great energy overhead of nodes. Therefore, some schemes have been suggested to reduce location updating control messages. Ye et al. [19] presented a Two-Tier Data Dissemination (TTDD) protocol in which each data source proactively constructs a grid structure enabling mobile sinks to continuously receive data on the move by flooding queries within a local cell only. The sink confines the destination area as it moves in order to broadcast its location information within the destination area only rather than to the entire network so as to reduce energy consumption of location updating message. Similarly, in [8], Wang et al. proposed Adaptive Local Update-based Routing Protocol (ALURP), which uses local flooding method to effectively update the location information of a mobile sink. However, there is substantial overhead for sink's location updating, especially when the sink moves at high speed. Shin and Kim [9] proposed a milestone-based predictive routing protocol that improves energy efficiency and prolongs the lifetime of networks. By introducing milestone node, the estimated sink's future location information is spread towards the nodes located in the vicinity of the recent trail of the sink by multihop relay by milestone node. The neighbors of these relay nodes can update their own “routing information” by overhearing, as a result, all local nodes can acquire the latest location information of the mobile sink. This protocol improves energy consumption and data packet delivery ratios. However, it still needs substantial overhead when sinks change their moving direction frequently. Shi et al. [20] proposed an efficient data-driven routing protocol with mobile sinks (DDRP). In order to reduce the protocol overhead for route discovery and maintenance caused by sink mobility while keeping high packet delivery, DDRP integrates data-driven routing and random walk routing in its implementation. Exploiting the broadcast feature of wireless medium, nodes overhear the data packets transmitted by their neighbors to learn fresh route information towards the sink. When no route to the mobile sink is known, random walk routing is adopted for data packet forwarding. DDRP can achieve lower protocol overhead and longer network lifetime. Fodor and Vidács [21] reduced communication overheads by proposing a restricted flooding method. Routes are updated only when topology changes. In [22], the authors utilize a logical coordinate system to infer distances and establish data reporting routing by greedily selecting the shortest path to the destination reference. It effectively reduces energy consumption. However, when changing its location, the mobile sink still needs to reestablish logical coordinate system.

### 2.3. Reduction of Time Delay of Data Reporting

The introduction of mobile sink may cause serious time delay problem; therefore, many researchers seek solutions to this kind of problems under time-sensitive application scenarios. Liang et al. [14] studied the network lifetime maximization problem for time-sensitive data gathering using a mobile sink with several constraints, such as the total travel distance and maximum distance between the sink's two sojourning locations. They presented a mixed integer linear programming solution to this multiple-constrained problem and proposed a heuristic solution. Xing et al. [6] proposed a rendezvous-based approach in which a subset of nodes serve as the rendezvous points (RPs) that buffer data originated from sources and transfer to MEs when they arrive. Taking data delivery deadline into account, RP-CP and RP-UG were proposed to facilitate reliable data transfers from RPs to MEs under the condition of significant unexpected delays in ME movement and network communication. Aioffi et al. [23] proposed the Minimum Wiener index Spanning Tree (MWST) as a routing topology for multiple base stations. A branch and bound algorithm for small-scale WSNs and a simulated annealing algorithm for large-scale WSNs are designed alternatively. The energy efficiency and packet delay attributes performance better than that of traditional minimum spanning tree.

In this paper, a location predictive and time adaptive data gathering scheme with mobile sink is proposed for wireless sensor networks. The trajectory of mobile sink can be a predefined circle, rectangle, or other geometric shapes depending on the deployed area, and the moving velocity of the sink is a constant. These two conditions make the mobile sink location predictable, which reduces the energy overhead for broadcasting location update messages of the mobile sink while maintaining low data transmission delay. When reporting or forwarding data to the mobile sink, sensors calculate the location of the mobile sink based on a loose time synchronization mechanism among sensor nodes and the mobile sink. Different from [9], in LPTA, the mobile sink needs no location updating message to inform sensor nodes of its latest location, which saves a lot of control overhead. The sink collects data from sensors only when it is dwelling at sojourn points. The sink dwelling time at sojourn points is dynamically adjustable, but unlike depending on local nodes density in [16], time adjustment method in our scheme is based on the number of historical data generated in each area, which is more applicable to real environments.

## 3. Network Model and Problem Statement

**Figure 1:**
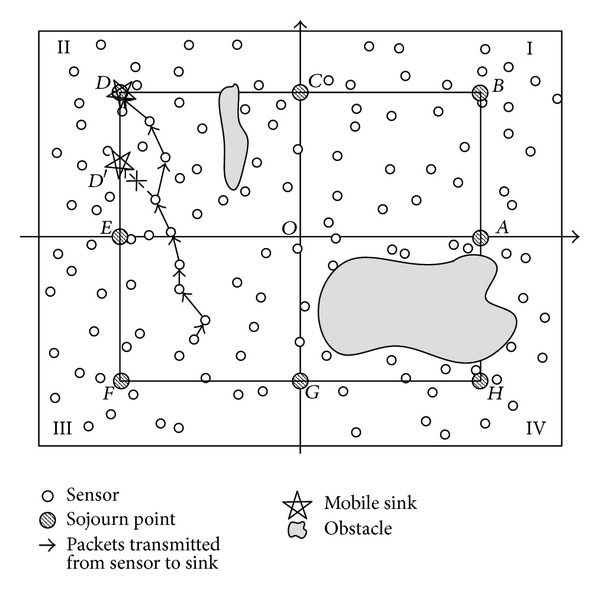
Network model of rectangle trajectory.

**Figure 2:**
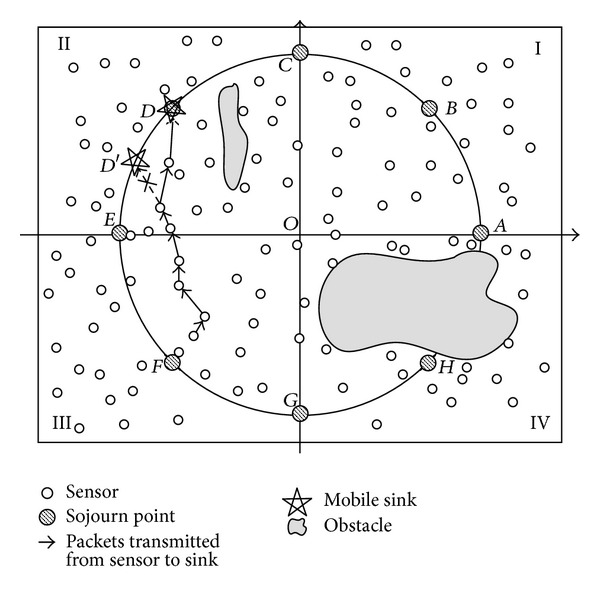
Network model of circle trajectory.

The network model is shown in Figure 1. *N* sensor nodes are deployed randomly in the network and one mobile sink gathers data from the whole network. All sensor nodes are quasi-stationary and location-aware (i.e., equipped with GPS-capable antennae). The mobile sink is not constrained by energy and can move at constant velocity. The whole network area is a *W* × *L* rectangle, and the mobile sink moves along a predefined trajectory. A predefined fixed trajectory is the base of location prediction, and many researchers have investigated the performance of different trajectory for data gathering protocols [10, 15, 24]. We use a rectangle of *w* × *l* and a circle with radius *R* as the examples of trajectories in our network as shown in Figures 1 and 2, respectively. The prediction of the location of mobile sink based on these two trajectories is simple and effective.

As described in Section 1, the frequentness and the number of the events of interest may be different in different part of the deployment area. And the network is deployed into two-dimensional Cartesian coordinate system. To simplify the related formulas and expressions, focus on the core algorithm of LPTA's dwelling time adjustment, and make it easily understood; we divide the network into four quadrants and its center is denoted as origin point *O*. The mobile sink turns off its radio transceiver while moving between two sojourn points and collects data from sensors only when it is dwelling at sojourn points. The number of sojourn points *n* is a multiple of four, and they are evenly distributed on the trajectory. Because the dwelling time of the mobile sink is adjusted based on the data generation portion of different region rather than different sojourn point, the number of sojourn points has no effect on the overhead of control message, and is only used for mobile gathering data in a predictive discrete manner. An anticlockwise rule is used to determine which quadrant a sojourn point belongs to; for example, point *A* belongs to quadrant I as shown in Figure 1. In Section 6, the simulation results under different sink's moving trajectory show that when sink moves along the rectangle trajectory, there is a better performance. Therefore, the location formulas of the mobile sink in our paper will be given based on a rectangle trajectory and other conditions such as circle trajectory can be acquired in a similar way.

Sensor nodes are able to communicate with the mobile sink by multihop relay. The nodes that can communicate directly with the sink within their communication radius *r* are one-hop neighbors of the mobile sink. For the sake of convenience, the main symbols used in this paper are listed in Notations Section.

There are two core problems to be solved in this paper. The first one is the moving strategy of the mobile sink. It includes the behavior of the mobile sink when the sink moves along the predefined trajectory and the dwelling time adjusting method in each quadrant. As illustrated in Figure 1, there may be obstacles in the monitoring area, for example, pools or swamps; we assume nodes deployed in these area are disabled to monitor the surroundings, which will cause the difference of data amount generating from each quadrant. Therefore, it is necessary to adjust the dwelling time of mobile sink to reside longer in the quadrant which generates more data packets. The mobile sink can be a quadcopter or an aircraft which is not influenced by these obstacles when moving along the trajectory. The second one is the data routing method for sensor nodes reporting data towards the mobile sink. Multihop relay communication method among nodes is adopted, as shown in Figure 1. Source node uploads data packets towards the mobile sink by multihop relay. Both of them will be stated in detail in the following section.

## 4. Sink Moving Strategy

In this section, we describe the data gathering process of mobile sink and introduce the dwelling time adaptive criteria. The data gathering process is periodically carried out by the mobile sink. During the periodical data gathering process, the mobile sink circles along the trajectory, stops at a sojourn point to collect sensed data, and then moves to the next sojourn point. To simplify our data gathering scheme, we divide the whole data gathering process into several time intervals, each of which is corresponding to a data gathering period (DGP). The mobile sink circles along the trajectory over and over in one data gathering period, and we further divide one data gathering period into multiple data gathering circles (DGPs). The detailed definition of data gathering period and data gathering circle are as follows.

- *Data Gathering Period (DGP)*. A DGP is defined as the process from the beginning of the sink entering the network to its leaving the network. During one DGP, the mobile sink carries out the data gathering algorithm which includes loose time synchronization phase, time adaptive data collection phase, and DGP ending declaration phase. One DGP is composed of multiple DGCs.
- *Data Gathering Circle (DGC)*. A DGC refers to the process of the sink moving along the trajectory, and backing to the initial point. For example, the sink starts from sojourn point *A*, moves along the trajectory, passes through sojourn points from *B* to *H*, and comes back to point *A* again, as shown in Figure 1. This process is a DGC.

The number of DGCs contained in one DGP depends on the application requirements and is limited by the available energy carried by the mobile sink. The higher the value, the better it is. Because at the beginning and the ending of one DGP, the mobile sink needs to broadcast a* HELLO* message and a* BYE* message, respectively, which will consume the energy of nodes. Considering realistic situation, it is set 10 in our simulation. Additionally, as the relevant formulas of sink's location is related to sink's moving trajectory, we only give the formulas hereinafter according to the rectangle moving trajectory; similar methods can be used to acquire the expression of other shapes.

### 4.1. Loose Time Synchronization

The mobile sink as well as every node in the network owns its own clock. At the beginning of one DGP, the mobile sink broadcasts a time synchronization message,* HELLO*, to achieve loose time synchronization among the mobile sink and all nodes in the network. Based on this synchronization, every node in the network can calculate the location of the mobile sink according to its local time information when uploading data packets to the mobile sink.

The loose time synchronization phase is the first phase during one DGP. When entering into the network, the mobile sink broadcasts a* HELLO* message to the whole network. The* HELLO* message consists of the starting location information *S*(*x*, *y*), current time *t*_0_, the moving velocity *V* of the mobile sink, the number of sojourn points *n* on the trajectory, the dwelling time at each sojourn point in quadrant *i*   (*i* ∈ {1,2, 3,4}) during the first DGC *T*_*s*_(*i*, 1), and the width and length of rectangle trajectory *w* and *l*. Every node changes its clock to *t*_0_ when it receives the* HELLO* message for the first time and then retransmits this message to its neighbors. Note that the parameters *T*_*s*_(*i*, 1) in the* HELLO* message are equal to each other, that is *T*_*s*_(1,1) = *T*_*s*_(2,1) = *T*_*s*_(3,1) = *T*_*s*_(4,1) = *T*_*s*_.

After the network achieved loose time synchronization, the mobile sink starts to collect data packets from the network. The time for loose time synchronization can be ignored since it is quite small compared with the time for one DGP.

### 4.2. Time Adaptive Data Collection

During the time adaptive data collection phase, the dwelling time is adjusted dynamically. In some application scenarios, there may exist obstacles in the network area; hence the data generated from each quadrant can be different greatly. According to the variation degree of *P*_data_(*i*, *k* − 1) and *P*_data_(*i*, *k*), the dwelling time *T*_*s*_(*i*, *k* + 1) in the (*k* + 1)th DGC at sojourn points is adjusted dynamically. In this way, the energy consumption of entire network can be further balanced.

As the data packets in each quadrant are generated in a random manner and transmitted by multihop relay to the mobile sink, the routing path for these packets generated in quadrant *i* will be longer than those in quadrant *j* (*i* ≠ *j*) where the mobile sink locates; therefore, the former will consume much more energy than the latter. To reduce the energy consumption caused by long distance data packets routing, after finishing each DGC, the mobile sink statistics the number of packets received from each quadrant and then calculates the proportion of these packets to the entire network data packets *P*_data1_, *P*_data2_, *P*_data3_, and *P*_data4_, accordingly. Depending on these proportions, the dwelling time of the mobile sink at sojourn points in each quadrant is adjusted dynamically, which makes the energy consumption in the network more balanced and the network lifetime extended.

The principle of adjusting the dwelling time *T*_*s*_(*i*, *k* + 1) in the (*k* + 1)th DGC is described in detail as follows.

In order to distinguish the quadrant in which a data packet is generated, source nodes append a 2-bit quadrant information to the head of the data packet before sending it to its next hop. The quadrant information can be calculated based on the sensor node location loc⁡(*x*_*i*_, *y*_*i*_) relative to the origin point *O*'s location information. Note that only the source nodes need to add their own quadrant information to the head of the data packets.

During one DGP, the mobile sink calculates the (*k* + 1)th DGC's dwelling time in each quadrant according to the proportions *P*_data1_, *P*_data2_, *P*_data3_, and *P*_data4_ in the (*k* − 1)th DGC and the proportions in the *k*th DGC. When the value *P*_change_(*k*, *k* − 1) is greater than the threshold value *T*_*h*_, the dwelling time in corresponding quadrant will be adjusted as *T*_*s*_(*i*, *k* + 1) = 4*P*_data*i*_ *T*_*s*_. The value of *P*_change_(*k*, *k* − 1) is calculated according to the following formula:

(1) Pchange(k,k−1) =∑i=14(Pdata(i,k)−Pdata(i,k−1))2.

*P*_change_ (*k*, *k* − 1) represents the variation degree of data generation proportion in different quadrant between two adjacent DGC. When this value is greater than the threshold *T*_*h*_, it means that the quantity of data packets generated in each quadrant has changed significantly, and the dwelling time needs to be adjusted. Under this circumstance, the mobile sink will broadcast a* UPDATE* message to all nodes in the network, which includes the adjusted dwelling time in each quadrant *T*_*s*_(*i*, *k* + 1). Otherwise, there is no necessity to modify the dwelling time, and the mobile sink maintains the dwelling time in each quadrant the same as the previous DGC.

### 4.3. DGP Ending Declaration

DPG ending declaration phase is the last phase in one DGP. At the beginning of the last DGC of one DGP, the mobile sink broadcasts a* BYE* message to all nodes in the network. The broadcasting of* BYE* message means that the current DGP is coming to an end, and the mobile sink will stop gathering data and leave the network. The* BYE* message consists of the time *T*_bl_, which is the time interval between current time and the mobile sink finishing current DGP. Instead of routing the data to the mobile sink, when receiving* BYE* messages, nodes will buffer the data sensed from surroundings after time *T*_bl_:

(2) $$\begin{array}{c} T_{\text{bl}}=nT_{s}+\frac{2\left(w+l\right)}{V}-2T_{\text{syn}}. \end{array}$$

*T*_syn_ is the time needed for the network to achieve loose time synchronization and, as to *T*_syn_, there is

(3) $$\begin{array}{c} T_{\text{syn}}\ll nT_{s}+\frac{2\left(w+l\right)}{V}. \end{array}$$

Therefore *T*_syn_ has little effect on *T*_bl_ and can be ignored in practical applications.

## 5. Data Reporting Process

In the network, source nodes transmit data packets to the mobile sink by multiple hops. The principle of selecting next hop is to make the path between a source node and the mobile sink approximately shortest. When nodes have data to report or relay, they need to calculate the mobile sinks current location based on their own clocks, which have been loosely synchronized to the mobile sink, and then choose one of its neighbors as the next hop. Using other existing routing protocols for data routing is also feasible. Our scheme focuses on sink's location prediction and its dwelling time adjustment. If there exists the concave obstacle which may result in data reporting failed, obstacle avoidance routing protocols can be adopted to relay the data packets to the mobile sink.

The time step *T*_step_ is the time interval for moving between two adjacent sojourn points. It is calculated by the following formula:

(4) $$\begin{array}{c} T_{\text{step}}=\frac{2\left(w+l\right)}{nV}. \end{array}$$

During the loose time synchronization phase, the mobile sink broadcasts a* HELLO* message to achieve loose time synchronization among all the nodes in the network. The parameter *T*_*s*_(*i*, 1) in the* HELLO* message is equal to each other; that is, *T*_*s*_(1,1) = *T*_*s*_(2,1) = *T*_*s*_(3,1) = *T*_*s*_(4,1) = *T*_*s*_. *T*_*s*_ is a constant value and keeps unchanging during a DGP.

**Figure 3:**
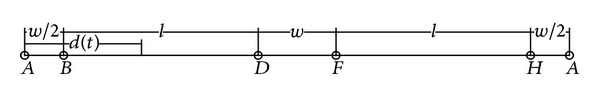
Mapping model of rectangle trajectory.

To determine the location of the mobile sink at time *t*, in this paper, we have the moving trajectory of the mobile sink map to a line model. In the model, the starting location of a DGP is chosen as the reference location, for example, point *A* in Figure 3. Corresponding to the rectangle trajectory illustrated in Figure 1, the line model is shown in Figure 3, in which the *d*(*t*) is the distance from the reference location *A* to the current location of mobile sink in the present DGC.

The moving time of the mobile sink in current DGC is denoted as *T*_*p*_, which is calculated by the following formula:

(5) $$\begin{array}{c} T_{p}=\left[\frac{t-t_{0}}{n\left(T_{\text{step}}+T_{s}\right)}\right]. \end{array}$$

Based on loose time synchronization, the location of the mobile sink can be calculated by the following formulas:

(1) If
  (6) $$\begin{array}{c} 0\leq T_{p}<\frac{nT_{s}\left(1,k\right)}{4}+\frac{w+l}{2V}, \end{array}$$
  then
  (7) $$\begin{array}{c} \text{Loc}\left(t\right)=\{\begin{array}{cc} \left(-\frac{l}{2},d\left(t\right)\right), & d\left(t\right)<\frac{w}{2},\\ \left(-\frac{l}{2}+\left(d\left(t\right)-\frac{w}{2}\right),\frac{w}{2}\right), & \text{others} \end{array} \end{array}$$
  *d*(*t*) can be calculated as below:
  (8) $$\begin{array}{c} T_{1}=\frac{\left(T_{p}-\left\lfloor T_{p}/\left(T_{\text{step}}+T_{s}\left(1,k\right)\right)\right\rfloor \left(T_{\text{step}}+T_{s}\left(1,k\right)\right)\right)}{\left(T_{s}\left(1,k\right)\right)} \end{array}$$
  and if ⌊*T*_1_⌋ = 0, then
  (9) $$\begin{array}{c} d\left(t\right)=V\left\lfloor \frac{T_{p}}{T_{\text{step}}+T_{s}\left(1,k\right)}\right\rfloor T_{\text{step}} \end{array}$$
  else
  (10) $$\begin{array}{c} d\left(t\right)=V\left(T_{p}-\left\lceil \frac{T_{p}}{T_{\text{step}}+T_{s}\left(1,k\right)}\right\rceil T_{s}\left(1,k\right)\right). \end{array}$$
(2) If
  (11) nTs(1,k)4+w+l2V ≤Tp<n(Ts(1,k)+Ts(2,k))4+2(w+l)2V,
  then
  (12) $$\begin{array}{c} \text{Loc}\left(t\right)=\{\begin{array}{cc} \left(-\frac{l}{2}+\left(d\left(t\right)-\frac{w}{2}\right),\frac{w}{2}\right), & d\left(t\right)<\frac{w}{2}+l\\ \left(\frac{l}{2},\frac{w}{2}-\left(d\left(t\right)-l-\frac{w}{2}\right)\right), & \text{others} \end{array} \end{array}$$
  *d*(*t*) can be calculated as below:
  (13) T2=(Tp−nTs(1,k)4−w+l2V−⌊Tp−nTs(1,k)/4−(w+l)/2VTstep+Ts(2,k)⌋×(Tstep+Ts(2,k)))  ×(Ts(2,k))−1
  and if ⌊*T*_2_⌋ = 0, then
  (14) d(t)=V(w+l2V+⌊Tp−nTs(1,k)/4−(w+l)/2VTstep+Ts(2,k)⌋×Tstep)
  else
  (15) d(t)=V(Tp−nTs(1,k)4−⌈Tp−nTs(1,k)/4−(w+l)/2VTstep+Ts(2,k)⌉Ts(2,k)).
(3) If
  (16) n(Ts(1,k)+Ts(2,k))4+2(w+l)2V≤Tp <n(Ts(1,k)+Ts(2,k)+Ts(3,k))4+3(w+l)2V,
  then
  (17) $$\begin{array}{c} \text{Loc}\left(t\right)=\{\begin{array}{cc} \left(\frac{l}{2},-d\left(t\right)+l+w\right), & d\left(t\right)<\frac{3w}{2}+l\\ \left(\frac{l}{2}-\left(d\left(t\right)-l-\frac{3w}{2}\right),-\frac{w}{2}\right), & \text{others} \end{array} \end{array}$$
  *d*(*t*) can be calculated as below:
  (18) T3=(Tp−n(Ts(1,k)+Ts(2,k))4−2(w+l)2V−⌊Tp−n(Ts(1,k)+Ts(2,k))/4−2(w+l)/2VTstep+Ts(3,k)⌋×(Tstep+Ts(3,k))) ×(Ts(3,k))−1
  and if ⌊*T*_3_⌋ = 0, then
  (19) d(t)=V(2(w+l)2V+⌊Tp−n(Ts(1,k)+Ts(2,k))/4−2(w+l)/2VTstep+Ts(3,k)⌋×Tstep)
  else
  (20) d(t)=V(Tp−n(Ts(1,k)+Ts(2,k))4−⌈Tp−n(Ts(1,k)+Ts(2,k))/4−2(w+l)/2VTstep+Ts(3,k)⌉×Ts(3,k)).
(4) If
  (21) n(Ts(1,k)+Ts(2,k)+Ts(3,k))4+3(w+l)2V ≤Tp<n(Ts(1,k)+Ts(2,k)+Ts(3,k)+Ts(4,k))4  +4(w+l)2V,
  then
  (22) Loc(t)={(l2−(d(t)−l−3w2),−w2),d(t)<3w2+2l(−l2,−w2+d(t)−2l−3w2),others
  *d*(*t*) can be calculated as below:
  (23) T4=(Tp−n(Ts(1,k)+Ts(2,k)+Ts(3,k))4−3(w+l)2V−⌊(Tp−n(Ts(1,k)+Ts(2,k)+Ts(3,k))4−3(w+l)2V)×(Tstep+Ts(4,k))−1⌋×(Tstep+Ts(4,k))) ×(Ts(4,k))−1
  and if ⌊*T*_4_⌋ = 0, then
  (24) d(t)=V(3(w+l)2V+⌊(Tp−n(Ts(1,k)+Ts(2,k)+Ts(3,k))4−3(w+l)2V)×(Tstep+Ts(4,k))−1⌋×Tstep)
  else
  (25) d(t)=V(Tp−n(Ts(1,k)+Ts(2,k)+Ts(3,k))4−⌈(Tp−n(Ts(1,k)+Ts(2,k)+Ts(3,k))4−3(w+l)2V)×(Tstep+Ts(4,k))−1⌉×Ts(4,k)).

We define ⌊*x*⌋ as the largest integer no more than *x*, ⌈*x*⌉ as the smallest integer no less than *x*, and [*x*1/*x*2] as the reminder of *x*1 divided by *x*2. To keep the formula as simple as possible, we require the starting point of data gathering to be on the intersection of *x* axis or *y* axis. Without loss of generality, we choose the location *A* as shown in Figure 1 as the starting point and deduce a series of formulas above.

**Figure 4:**
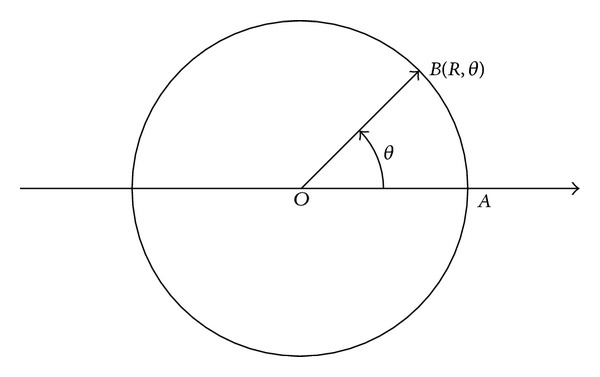
The model of polar system.

As described in Section 3, the moving trajectory can be a rectangle or circle. When the sink's moving trajectory is a circle, the time-location formulas can be acquired in a similar way. The difference is that the moving trajectory of the mobile sink is mapped into a polar coordinate system, because of calculation convenience.  Figure 4 is a model of polar system, assuming the mobile sink starts its data gathering process from point *A* at time *t*_0_ and reaches point *B* at time *t*, the arc length of $\overset{⌢}{AB}$ is *Rθ* (0 ≤ *θ* < 2*π*). When the sink moves at speed *V* in the network, there is *V*(*t* − *t*_0_) = *Rθ*. The corresponding polar coordinate of point *B* is (*R*, *θ*).

Similar to rectangle trajectory, based on loose time synchronization, the location of the mobile sink can be acquired accurately when the trajectory is a circle. For example, when the mobile sink locates at the first quadrant, then *T*_*p*_ meets 0 ≤ *T*_*p*_ < (*n*/4)*T*_*s*_(1, *k*) + 2*πR*/4*V*, and the polar angle of the sink is calculated by the following formulas:

If

(26) $$\begin{array}{c} \frac{T_{p}-\left\lceil T_{p}/\left(T_{\text{step}}+T_{s}\left(1,k\right)\right)\right\rceil \left(T_{\text{step}}+T_{s}\left(1,k\right)\right)}{T_{s}\left(1,k\right)}\rceil =0, \end{array}$$

then

(27) $$\begin{array}{c} \theta =\frac{2\pi }{n}\left\lceil \frac{T_{p}}{T_{\text{step}}+T_{s}\left(1,k\right)}\right\rceil , \end{array}$$

else

(28) $$\begin{array}{c} \theta =\frac{V}{R}\left(T_{p}-\left\lceil \frac{T_{p}}{T_{\text{step}}+T_{s}\left(1,k\right)}\right\rceil T_{s}\left(1,k\right)\right). \end{array}$$

When the sink locates at other quadrants, the corresponding location information can be obtained in a similar manner.

According to the calculated location information, source nodes upload their sensed data to the mobile sink by multihop communication. When events occur in the monitoring area, the sensors outside the communication range of the mobile sink route data packets to their next hop directly. It is unnecessary to judge the current state of the mobile sink, that is, moving between sojourn points or gathering data packets at a sojourn point. Only the neighbor nodes of the mobile sink need to judge the state of the mobile sink. If the mobile sink is moving between sojourn points, the neighbor nodes have to wait for a period of time *T*_*wl*_ until the former arrives at its next sojourn point. Otherwise, they transmit the data packet to the mobile sink directly. For instance, as shown in Figure 1, we assume the current location of the mobile sink is point *D*; if the events occur in quadrant III, then data packets can be routed along the shortest routing path to point *D*. When the data packets reach the neighbor node of the mobile sink, it will judge the state of mobile sink according to its local time clock. If the time of the node meets *t*_0_ + *k*(*T*_step_ + *T*_*s*_(*i*, *k*)) < *t* < *t*_0_ + *k*(*T*_step_ + *T*_*s*_(*i*, *k*)) + *T*_*s*_(*i*, *k*), which means the mobile sink is still gathering data at the sojourn point *D*, then this one-hop neighbor node of the sink transmits the data packets directly to the mobile sink; otherwise, for example, the sink is now located at *D*′, it needs to wait time *T*_*wl*_ and then transmit the data packet to the mobile sink. The time *T*_*wl*_ is calculated by the following formula:

(29) $$\begin{array}{c} T_{wl}=t_{0}+\left(k+1\right)\left(T_{\text{step}}+T_{s}\left(i,k\right)\right)-t. \end{array}$$

During routing data packets to the mobile sink, hop-by-hop acknowledgement mechanism is applied to ensure the data transmission rate, that is, if the receiver* Node2* gets the data packets from sender* Node1*, it will reply with an* ACK* message to* Node1*. If* Node1* does not receive the* ACK* message from its next hop node* Node2* within time *T*,* Node1* considers that the packet transmission is failed and will cache the data packets and wait for a random time and then retransmit the packets to its next hop again. We assume that *T* is equal to the propagation time of a packet between two farthest nodes of the network.

## 6. Performance Evaluation

In this section, we evaluate the performance of our scheme through extensive simulations. In addition to the proposed scheme, we implemented the* Adaptive* [16] for comparison. The reason is that* Adaptive* is also a discrete data gathering protocol. Besides, the mobile sink in* Adaptive* dwells at sojourn points on the predefined trajectory for data collection, and the dwelling time is dynamically adjusted. Two performance metrics, energy consumption and data delivery latency, are investigated. Energy consumption is the average energy that is consumed by nodes during one DGP. And data delivery latency is the time interval from a message creation to the mobile sink receiving it.

### 6.1. Simulation Environment

We implement our proposed scheme in MatLab. In our simulation, the deployment area has a 500 m × 500 m square sensing field and sensor nodes are randomly deployed in the network, that is, area length *L* equals its width *W*. The communication range of the nodes and the mobile sink is set to 60 m. The mobile sink moves along the predefined trajectory for 10 circles in every DGP, and in every DGC, 5% of sensor nodes act as source nodes, which send messages toward the mobile sink continually when the sink is dwelling at sojourn points.

Different simulation environments with varying sink moving trajectory, number of nodes *N*, and mobile sink speed *V* are studied. We set the trajectory as circle and rectangle, the length of side or radius of trajectory is set as *L*/4, *L*/2, 3*L*/4, and *L*. And we varied *N* from 800 to 1200, *V* from 4 m/s to 20 m/s. Several groups of simulation experiments are carried out. The threshold of adjusting dwelling time *T*_*h*_ is set as 0, 0.25, 0.5, 0.75, and 1. *T*_*h*_ = 0 means the dwelling time of the mobile sink needs to be changed if the proportion of packets amount quantity generated from every quadrant is not exactly the same as previous DGC, while *T*_*h*_ = 1 means the dwelling time keeps unchanging during one DGP.

### 6.2. Simulation Results with Varying Sink Moving Trajectory

**Figure 5:**
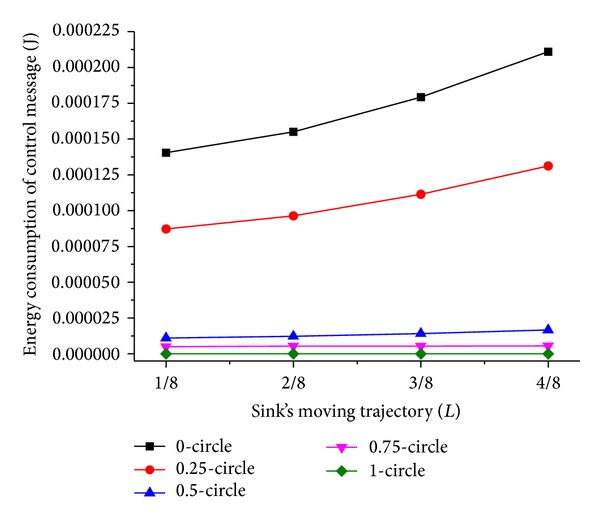
Energy consumption of control message with circle trajectory.

**Figure 6:**
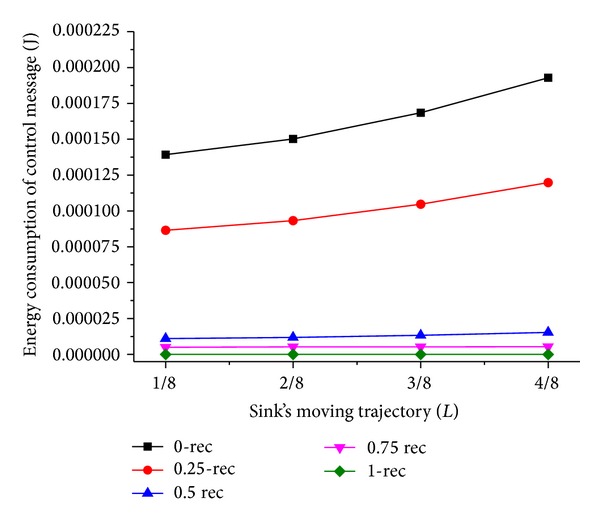
Energy consumption of control message with rectangle trajectory.

**Figure 7:**
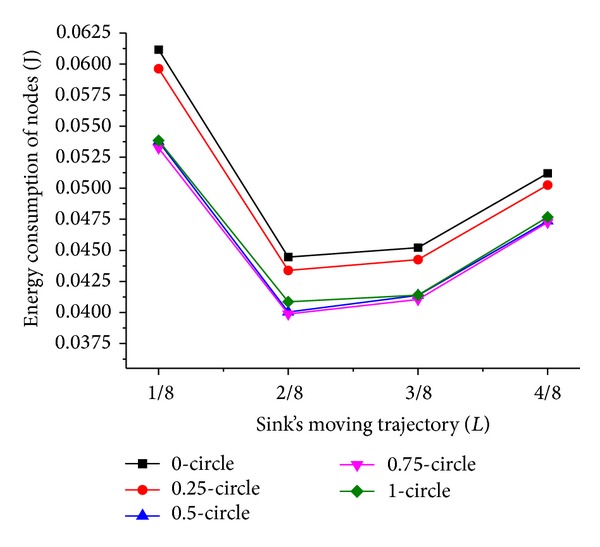
Energy consumption of nodes with circle trajectory.

**Figure 8:**
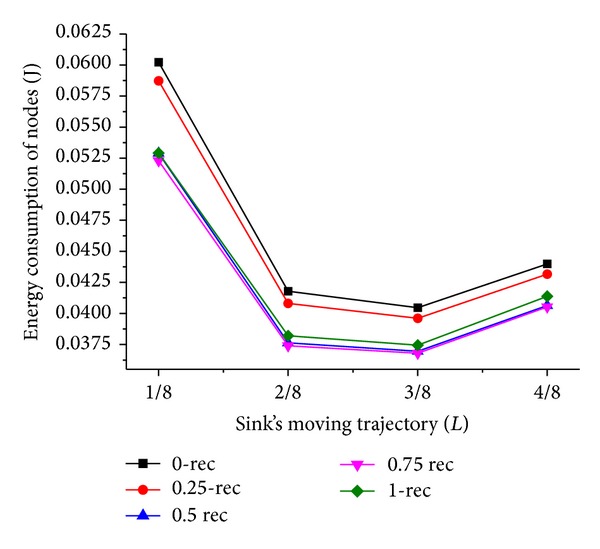
Energy consumption of nodes with rectangle trajectory.

Now we discuss the influence of sink moving trajectory on network performance. We compare two scenarios of the trajectory; they are rectangle and circle, and the mobile sink moves along the predefined trajectory for one DGP. The simulation results are shown in Figures 5, 6, 7, and 8.

Comparing Figures 5 and 7 with Figures 6 and 8, respectively, it is noticed that the energy consumption of nodes is lower when the moving trajectory is rectangle than that of circle when the deployment area is rectangle. The sharp of sink's moving trajectory has influence on energy consumption of nodes even if the path is predefined. In the following simulation, the moving trajectory of the mobile sink is rectangular which is similar to network deployment area.

As shown in Figures 7 and 8, the energy consumption is the lowest while mobile sink moves along the track with *L*/4 and 3*L*/4 length of side or radius. It is different from the theory proposed in [15] that peripheral movement is the best strategy, because the ideal load-balanced routing is hard to satisfy. Under the condition of a certain trajectory, there is an outstanding performance when *T*_*h*_ is less than 1; this is because the dynamic adjustment of dwelling time is beneficial to the performance of network. Besides, when *T*_*h*_ = 0.75, as shown in Figure 5, the energy consumption of control message is very low. This is because there is an appropriate tradeoff between the control overhead and the balanced energy consumption among different quadrants when *T*_*h*_ = 0.75. In contrast, the dwelling time adjustment frequency is too high when *T*_*h*_ = 0, resulting in much energy overhead. When *T*_*h*_ = 1, the energy consumption of control message equals 0, which means there is no dwelling time adjustment and the energy consumption among nodes is not well balanced.

### 6.3. Simulation Results with Varying Number of Sensor Nodes

**Figure 9:**
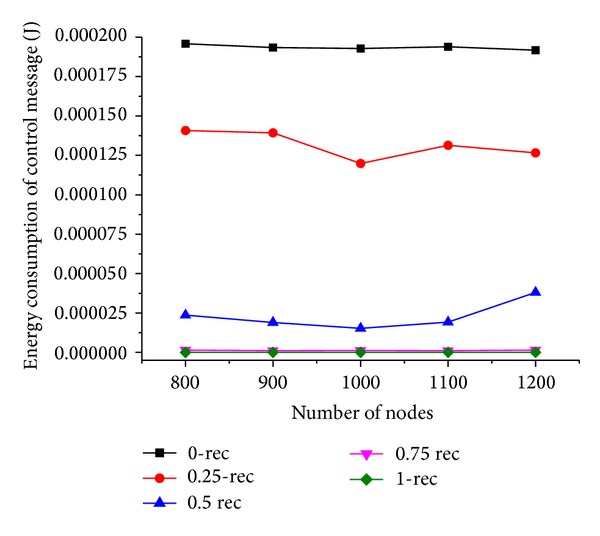
Energy consumption of control message under varying number of nodes.

**Figure 10:**
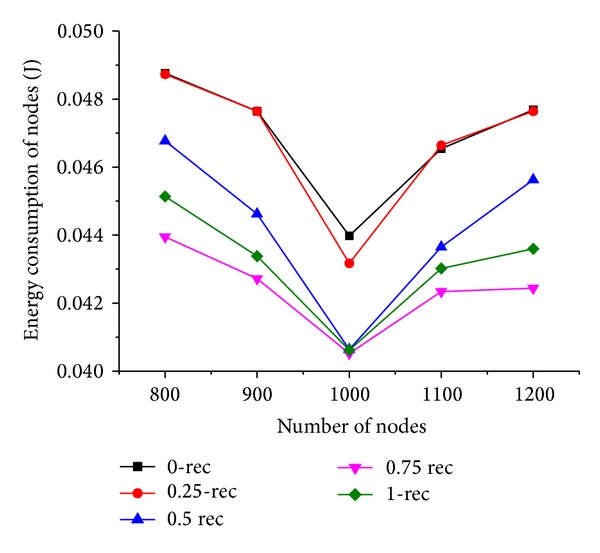
Energy consumption of nodes under varying number of nodes.

Now we discuss the performance of our scheme by setting the number of sensor nodes *N* varying from 800 to 1000 when the moving trajectory is rectangle. The simulation results are shown in Figures 9 and 10.

As illustrated in Figure 10, the energy consumption of nodes decreases first with increased number of nodes and then increases when *N* is more than 1000. It is because, with the increase of *N*, the number of selectable next hop neighbors increases, as a result, the hop distance and the routing path between the mobile sink and source nodes are improved, which results in less energy consumption for relaying the same quantity of data packets. When *N* is more than 1000, the increase of the number of source nodes causes more packets generating in the network, which results in more energy consumption of nodes when *N* increases. The performance is outstanding compared to the others when *T*_*h*_ is 0.75, and the reason is the same as explained in Section 6.2.

### 6.4. Simulation Results with Varying Sink Speed

**Figure 11:**
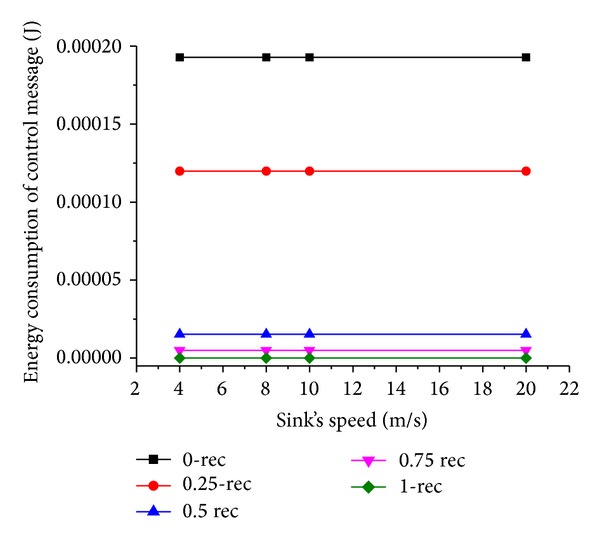
Energy consumption of control message under varying sink speed.

**Figure 12:**
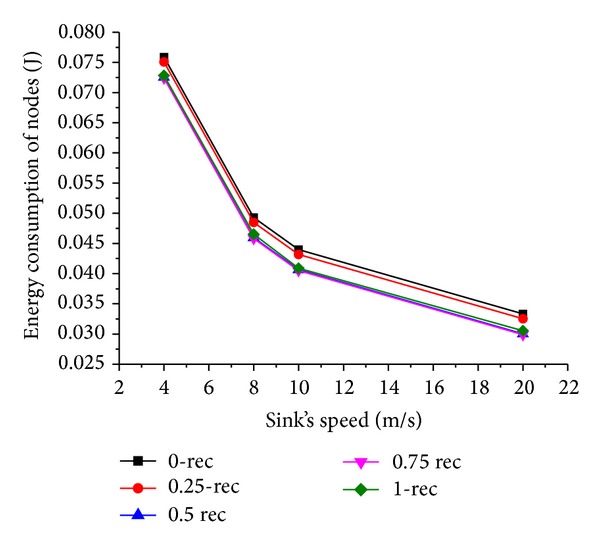
Energy consumption of nodes under varying sink speed.

Now we evaluate the network performance when sink's moving speed *V* varies from 4 m/s to 20 m/s. The results are shown in Figures 11 and 12.

It is noticed that as the mobile sink speed goes up, the energy consumption decreases. This is because with the increase of sink speed, the time the mobile sink spending for moving between two adjacent sojourn points decreases, and the mobile sink can receive the data packets timely from sensor nodes. This results in less energy consumed.

### 6.5. Simulation Results of Data Transmission Delay and Energy Consumption

We simulated our proposed scheme, as well as the adaptive algorithm and constant algorithm described in the adaptive sink mobility scheme proposed by Kinalis et al. [16], to evaluate the performance of data delivery latency and energy consumption by varying sink's moving speed.

**Figure 13:**
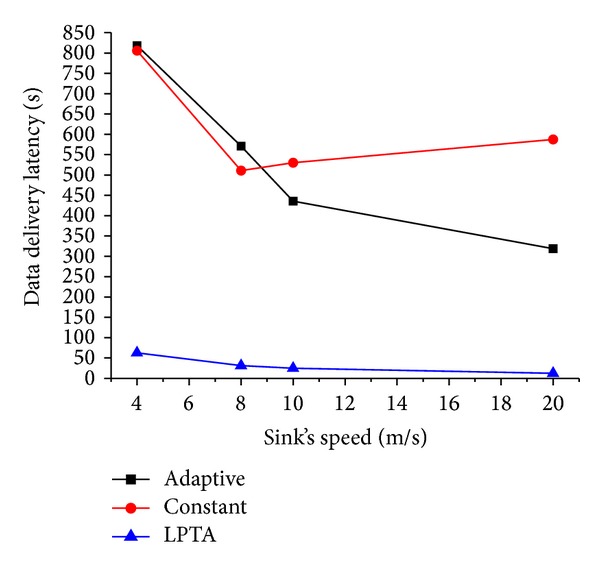
Data transmission latency under varying sink speed.

The results are shown in Figure 13. Our LPTA scheme outperforms the Adaptive scheme in the attribute of latency. The reason is that in LPTA, the latency is mainly caused by the mobile sink turning off its communication model when moving between two adjacent sojourn points. But in adaptive and constant algorithms, the sink has to traverse all vertexes, which results in large time delay. Besides, the increase of speed is beneficial for our LPTA. It is because the time needed decreases for moving the same distance with higher moving speed. Hence, the data transmission delay significantly reduced with the increase of sink speed.

**Figure 14:**
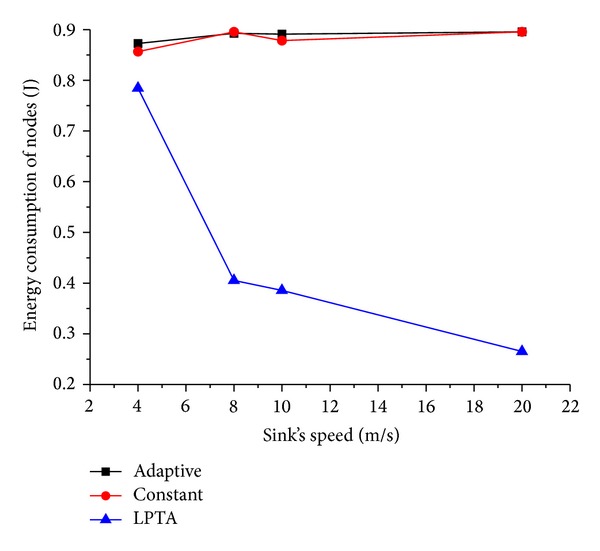
Energy consumption under varying sink speed.

Figure 14 shows the performance of energy consumption with the change in velocity. When the sink moving speed is relatively small,* adaptive* algorithm performance is almost the same as our LPTA. However, with the increasing of sink's moving speed, the energy consumption of* adaptive* and* constant* algorithms are much more than LPTA. It is because with the increasing of sink's speed, the time the mobile sink is spending for moving between two adjacent sojourn points decreases. As a result, the mobile sink can receive the data packets timely from sensor nodes, and the hops and routing path are shortened. Therefore, the energy consumption decreases accordingly.

## 7. Conclusion

In this paper, we propose a location predictive and time adaptive data gathering scheme with one mobile sink. Based on the loose time synchronization, nodes can calculate the latest location information of the mobile sink. Therefore, source nodes are able to route data packets timely to the mobile sink by multihop relay. As a result, the energy overhead for updating sink's location is largely reduced. Along with the location predictive algorithm, this study also describes a dwelling time adjustment method for the mobile sink to efficiently balance the energy consumption among nodes. Simulation results show that the proposed data collection scheme provides improved performance on time latency and energy consumption compared to* adaptive *algorithm. However, as described in Section I, the environments of the deployment area are often harsh and involve obstacles. When some sojourn points happen to be on huge obstacles, it may have bad effect on data packets uploading to the mobile sink. Under this condition, when the mobile sink dwells on these sojourn points, data packets have to be uploaded for many times and even be lost, which will degrade the energy consumption performance and packet delivery ratio. For future research, we plan to enhance the LPTA scheme by considering huge obstacles avoidance to further improve the performance of data gathering algorithm.

## Notations

*T*_*s*_(*i*, *k*): The dwelling time of mobile sink at each sojourn point in quadrant *i* during the *k*th DGC in current DGP
*T*_syn_: The time needed for the network to achieve time synchronization
*P*_data_(*i*, *k*): The proportion of the collected data from quadrant *i* to the whole network during the *k*th circle
*T*_*p*_: The time since the beginning of present DGC
*T*_bl_: The time before the mobile sink leaving the network in present DGP
*r*: The communication radius of nodes and the mobile sink
*R*: The radius of the circle moving trajectory
*w*/*l*: The width/length of the rectangle moving trajectory
*n*: The number of sojourn points
*N*: The number of sensor nodes
*V*: The speed of the mobile sink.
